# Nanomedicine against oxaliplatin-induced peripheral neuropathy: hypotheses and research perspectives on ion channels and immune microenvironment

**DOI:** 10.1186/s43046-026-00355-w

**Published:** 2026-05-11

**Authors:** Yuan Gao, Pankui Li

**Affiliations:** 1 First Clinical College of Ningxia Medical University, Ningxia, China; 2 General Hospital of Ningxia Medical University, Yinchuan, China

**Keywords:** Oxaliplatin, Chemotherapy-induced peripheral neuropathy, Dorsal root ganglion, Ion channels, Nanomedicines, Salidroside, Spatial transcriptomics

## Abstract

Oxaliplatin serves as a cornerstone chemotherapeutic agent for solid tumors (e.g., colorectal and gastric cancers); however, its associated dose-limiting peripheral neuropathy (OIPN) severely compromises patients’ quality of life and treatment completion rates. OIPN manifests as acute cold-induced paresthesia and chronic cumulative sensory neuropathy, with complex, incompletely elucidated pathophysiological mechanisms. This review systematically summarizes the core molecular mechanisms of OIPN, focusing on: (1) sensitization of transient receptor potential vanilloid/ankyrin channels (TRPV1/TRPA1), (2) dysregulated expression and function of voltage-gated sodium channels (NaV1.7, NaV1.8), and (3) the critical role of p38 mitogen-activated protein kinase (p38-MAPK) pathway activation in neuronal hyperexcitability and pain signal transduction within dorsal root ganglion (DRG) sensory neurons. Additionally, we delve into dynamic alterations of the DRG immune microenvironment (notably macrophages and T cells) during OIPN initiation/progression, as well as their crosstalk with neurons. To address the clinical dilemma of limited effective preventive/therapeutic approaches, this review outlines limitations of current strategies and highlights the advantages of nanotechnology-based drug delivery systems in enhancing neuroprotective agent bioavailability and enabling targeted delivery. Finally, we hypothesize the integration of salidroside (a natural product with anti-inflammatory/antioxidant properties) with nanotechnology, and propose leveraging cutting-edge tools (spatial transcriptomics, single-cell RNA sequencing) to elucidate its potential mechanistic action in OIPN—providing a theoretical hypothesis and exploratory research directions for precision OIPN prevention and treatment, in the absence of any empirical evidence for salidroside’s efficacy in this specific pathological context.

## Introduction: oxaliplatin and peripheral neuropathy—an unresolved clinical challenge

Oxaliplatin, a third-generation platinum-based compound, exerts potent antitumor activity by forming intra- and inter-strand DNA crosslinks—adducts that block DNA replication and transcription. It has thus emerged as a core component of standard chemotherapy regimens for high-prevalence malignancies including colorectal and gastric cancers [1] However, up to 85–95% of patients develop varying degrees of peripheral neurotoxicity following oxaliplatin treatment, i.e., oxaliplatin-induced peripheral neuropathy (OIPN), which has become the dose-limiting toxicity (DLT) that restricts its clinical utility [2, 3].

OIPN exhibits a unique biphasic clinical manifestation: (1) Acute, reversible neuropathy: Typically onset within hours following infusion, it presents as transient paresthesia and hypoesthesia in distal extremities (especially hands, feet, and perioral regions), which are markedly triggered or exacerbated by cold exposure [4, 5]. (2) Chronic, cumulative neuropathy: Gradually developing with increasing chemotherapy cycles and cumulative doses, it is characterized by progressive numbness, hypoalgesia, fine motor dysfunction (e.g., difficulty buttoning clothes), and in severe cases, persistent pain and ataxia. Notably, this chronic form exhibits slow recovery, with irreversible symptoms in some patients [6]. Such chronic sensory neuropathy significantly impairs patients’ quality of life and functional status, and may lead to chemotherapy dose reduction, delay, or even discontinuation—ultimately compromising tumor treatment efficacy and patient prognosis [7].

Despite the widespread recognition of OIPN’s clinical significance in humans, its exact pathophysiological mechanisms remain incompletely elucidated in the clinical setting, resulting in the absence of evidence-based effective preventive and therapeutic agents for clinical practice in human patients. Current strategies—such as calcium-magnesium infusions, duloxetine, and pregabalin—are primarily symptomatic and supportive, with limited efficacy and associated adverse effects [8]. Thus, deciphering the molecular and cellular mechanisms of OIPN in depth and developing novel, effective preventive/therapeutic strategies accordingly represent a critical scientific challenge urgently requiring resolution in the fields of oncological supportive care and neuroscience.

Traditional studies have proposed that OIPN primarily arises from direct damage to sensory neurons in the dorsal root ganglion (DRG)—a key aggregation of primary sensory neuron somata. The DRG is characterized by a relatively underdeveloped blood-nerve barrier (BNB), which renders it susceptible to oxaliplatin accumulation [9]. In recent years, substantial advances have been made in relevant research, shifting the focus from isolated neuronal toxicity to the complex “neuron-glia-immune” crosstalk network. This review aims to systematically summarize the latest progress in deciphering the molecular mechanisms of OIPN, with a particular emphasis on ion channel dysfunction, activation of key signaling pathways, and immune microenvironment dysregulation. Building on these mechanisms, we further discuss the potential and research directions of novel intervention strategies, featuring nanotechnology as the delivery carrier and natural bioactive ingredients as the core.

This study is structured as a narrative review, which systematically synthesizes and integrates existing preclinical and clinical literature to delineate the molecular mechanisms underlying OIPN, critically evaluate current therapeutic limitations, and discuss the potential of nanomedicine-based intervention strategies—with a focus on hypothesis-driven exploration of salidroside as a candidate neuroprotective agent.

Literature Search Strategy: (1) Databases consulted: PubMed, Scopus, Web of Science, and Embase—four core databases widely recognized in the fields of oncology, neuroscience, and nanomedicine to ensure comprehensive coverage of relevant literature. (2) Search terms and keywords: A combination of MeSH terms and free-text keywords, including: (“oxaliplatin-induced peripheral neuropathy” OR “OIPN”) AND (“ion channels” OR “TRPV1” OR “TRPA1” OR “NaV1.7” OR “p38-MAPK”) AND (“dorsal root ganglion” OR “DRG immune microenvironment” OR “macrophages” OR “T cells”) AND (“nanomedicines” OR “nanoparticles” OR “targeted drug delivery”) AND (“salidroside” OR “natural neuroprotective agents”). 3.Time frame: Literature published between January 2010 and April 2025 was included to prioritize recent advances while ensuring coverage of foundational studies on OIPN mechanisms and nanomedicine development. 4. Inclusion and exclusion criteria: Included studies were peer-reviewed original research articles (preclinical in vitro/in vivo studies, clinical observational trials) and high-quality narrative/systematic reviews focusing on OIPN’s molecular mechanisms, nanomedicine-based delivery systems, or the pharmacological effects of salidroside in neurological disorders. Excluded studies were non-English publications, conference abstracts, case reports, and research unrelated to the core themes of OIPN, ion channels, DRG immune microenvironment, or nanomedicine-salidroside integration.

## Core molecular mechanisms of oxaliplatin-induced peripheral neuropathy

### Dysfunction of sensory neuronal ion channels: the cornerstone of electrophysiological disorders

The function of sensory neurons is highly dependent on the expression and activity of ion channels in their membranes. A growing body of evidence indicates that oxaliplatin can rapidly and specifically alter the function of multiple ion channels in DRG neurons, which is the direct cause of acute abnormal discharge and chronic hyperexcitability.

#### Sensitization of transient receptor potential (TRP) channels: key mediators of cold and mechanical pain

TRPV1 and TRPA1 are non-selective cation channels predominantly expressed in small-diameter, peptidergic (e.g., calcitonin gene-related peptide [CGRP]-positive) nociceptive sensory neurons. They are activated by distinct stimuli—TRPV1 by capsaicin and heat (> 43 °C), and TRPA1 by mustard oil, cold (< 17 °C), and reactive oxygen/nitrogen species (ROS/RNS)—and play a central role in pain perception [10, 11].

**Fig. 1:**
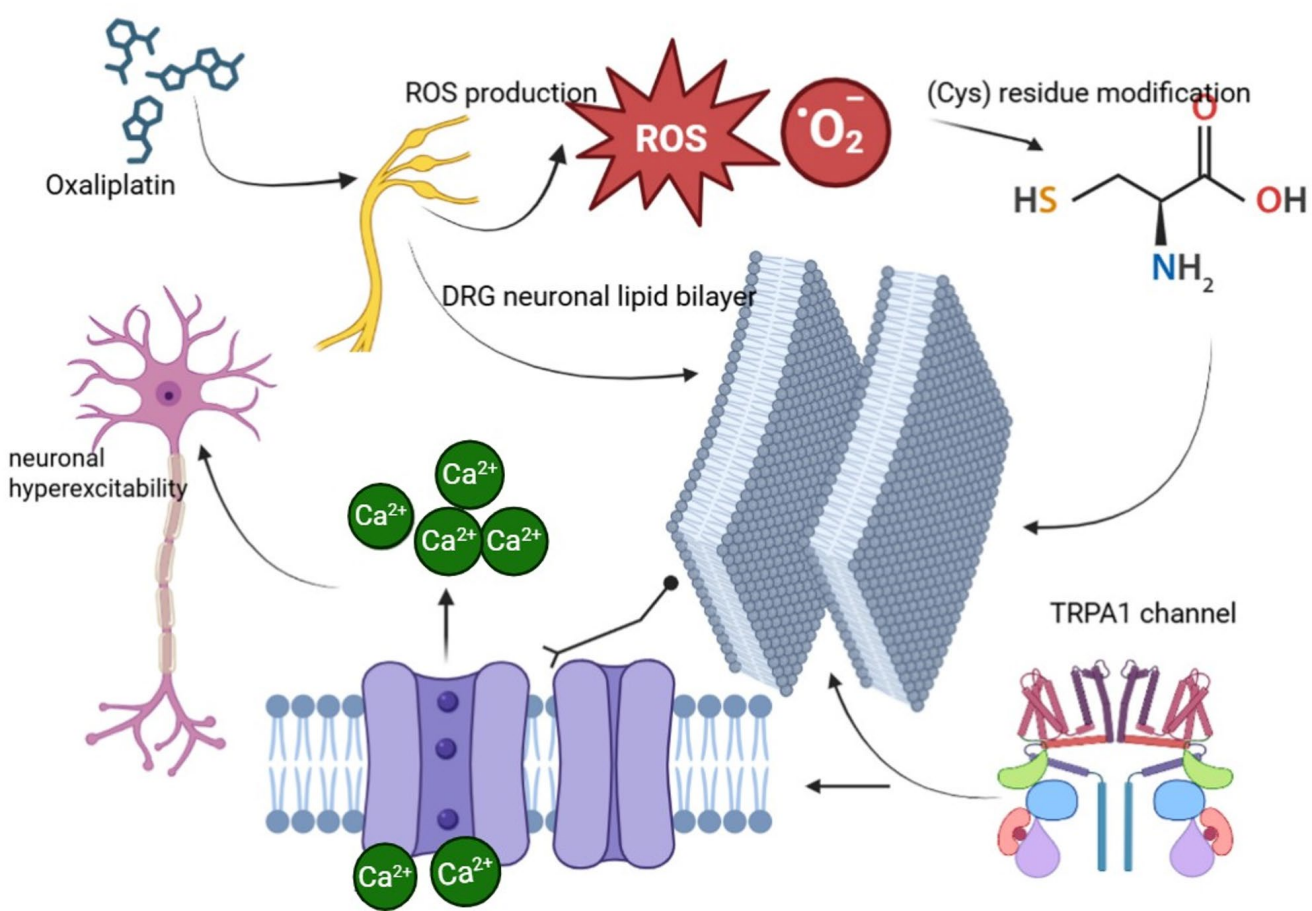
This schematic delineates a core pathophysiological cascade mediating oxaliplatin-induced peripheral neuropathy in dorsal root ganglion (DRG) neurons. 1. Oxaliplatin initiates reactive oxygen species (ROS) production (encompassing •O₂⁻ and H₂O₂, as labeled) in DRG neurons upon exposure. 2. The generated ROS mediates cysteine (Cys) residue modification of a membrane ion channel (localized on the DRG neuronal lipid bilayer; TRPA1 channel is also depicted in the adjacent membrane domain)0.3. This post-translational modification induces channel opening, which facilitates robust Ca²+ flux (inward calcium ion influx) across the DRG neuronal membrane. 4. Persistent Ca²+ influx ultimately elicits neuronal hyperexcitability—a critical pathophysiological event that contributes to the development of oxaliplatin-associated peripheral neuropathic phenotypes

Acute cold pain induced by oxaliplatin is its most characteristic symptom. Studies have demonstrated that oxaliplatin and its metabolites can directly or indirectly induce oxidative modification of specific cysteine residues in the TRPA1 channel, leading to its abnormal sensitization and increased open probability [12, 13]. In OIPN animal models, DRG neurons exhibit enhanced responsiveness to TRPA1 agonists, with increased action potential firing triggered by cold stimulation (Fig. 1); conversely, genetic knockout or pharmacological inhibition of TRPA1 significantly attenuates oxaliplatin-induced cold hyperalgesia but exerts minimal effects on mechanical hyperalgesia [14, 15]. This has established TRPA1 as a central mediator of acute cold pain in OIPN. TRPV1 is also implicated in OIPN, as oxaliplatin treatment enhances the responsiveness of DRG neurons to TRPV1 agonists [16]. The underlying mechanism may involve oxidative stress and activation of downstream kinases. Although TRPV1 is less specific than TRPA1 in mediating cold pain, its sensitization may contribute to generalized thermal hyperalgesia and spontaneous pain.

As the primary cold-sensing ion channel in human sensory neurons, transient receptor potential melastatin 8 (TRPM8) is a core mediator of physiological cold perception and pathological cold allodynia in OIPN, a defining feature of oxaliplatin-induced neuropathy [17]. Unlike rodent models where TRPA1 is the major cold-sensing effector, TRPM8 drives cold signal transduction in human DRG nociceptive and non-nociceptive sensory neurons, with its activation threshold matching the range of cold temperatures that trigger OIPN acute symptoms (10–28 °C), making it a species-specific key player in human OIPN cold hypersensitivity [17, 18].

Oxaliplatin modulates TRPM8 function and expression at both post-translational and transcriptional levels to induce its pathological sensitization. Oxaliplatin-induced ROS production in DRG neurons mediates covalent modification of key cysteine and lysine residues in the TRPM8 channel pore and transmembrane domains, increasing channel open probability and calcium influx in response to mild cold stimulation [19, 20]. Additionally, oxaliplatin-activated p38-MAPK signaling in DRG neurons drives transcription factor (ATF2/CREB)-dependent upregulation of TRPM8 mRNA and protein expression, further amplifying cold signal transduction [21]. These dual changes—sensitization and upregulation—synergistically enhance human DRG neuron responsiveness to cold stimuli, forming a critical ionic basis for OIPN-specific cold allodynia [22, 23].

Notably, TRPM8-mediated cold hypersensitivity is not an isolated event but is integrally embedded within the broader OIPN pathophysiological network, with extensive cross-regulation with other core pathological mediators. TRPM8 activity is synergistically enhanced by TRPA1-mediated calcium influx and NaV1.8/NaV1.7-dependent neuronal hyperexcitability, creating a positive feedback loop that amplifies cold-induced abnormal action potential firing [23]. Furthermore, pro-inflammatory cytokines (TNF-α, IL-1β) released from M1-polarized macrophages in the remodeled DRG immune microenvironment directly phosphorylate TRPM8 via p38-MAPK, further sensitizing the channel and linking immune dysregulation to cold hypersensitivity. This multi-level crosstalk positions TRPM8 at the intersection of ion channel dysfunction, signaling pathway activation, and neuro-immune crosstalk in OIPN.

Targeting TRPM8 holds significant theoretical potential for alleviating OIPN-specific cold allodynia, the most clinically distressing symptom of oxaliplatin-induced neuropathy. Selective TRPM8 antagonists (e.g., AMG517, PF-05105679) have demonstrated efficacy in preclinical models of peripheral neuropathic cold pain by blocking cold signal transduction without impairing normal thermal sensation [24, 25]. Combinatorial targeting of TRPM8 and TRPA1/NaV1.8 may yield synergistic effects for OIPN, addressing both human and rodent cold-sensing pathways and acute neuronal hyperexcitability. However, direct preclinical in vivo evidence and clinical trial data validating TRPM8-targeted agents in OIPN models and human patients remain lacking, and critical questions regarding optimal dosing, tissue selectivity, and potential off-target effects on thermoregulation require further investigation.

#### Functional upregulation of voltage-gated sodium channels (NaV): amplifiers of neuronal hyperexcitability

Voltage-gated sodium channels (NaV) are responsible for the initiation and propagation of action potentials. Among sensory neurons, NaV1.7, NaV1.8, and NaV1.9 are the predominant subtypes. Specifically, NaV1.7 sets the threshold for action potential generation, while NaV1.8 mediates the majority of current during the rising phase of action potentials and is critical for the conduction of nociceptive signals [26].

**Fig. 2:**
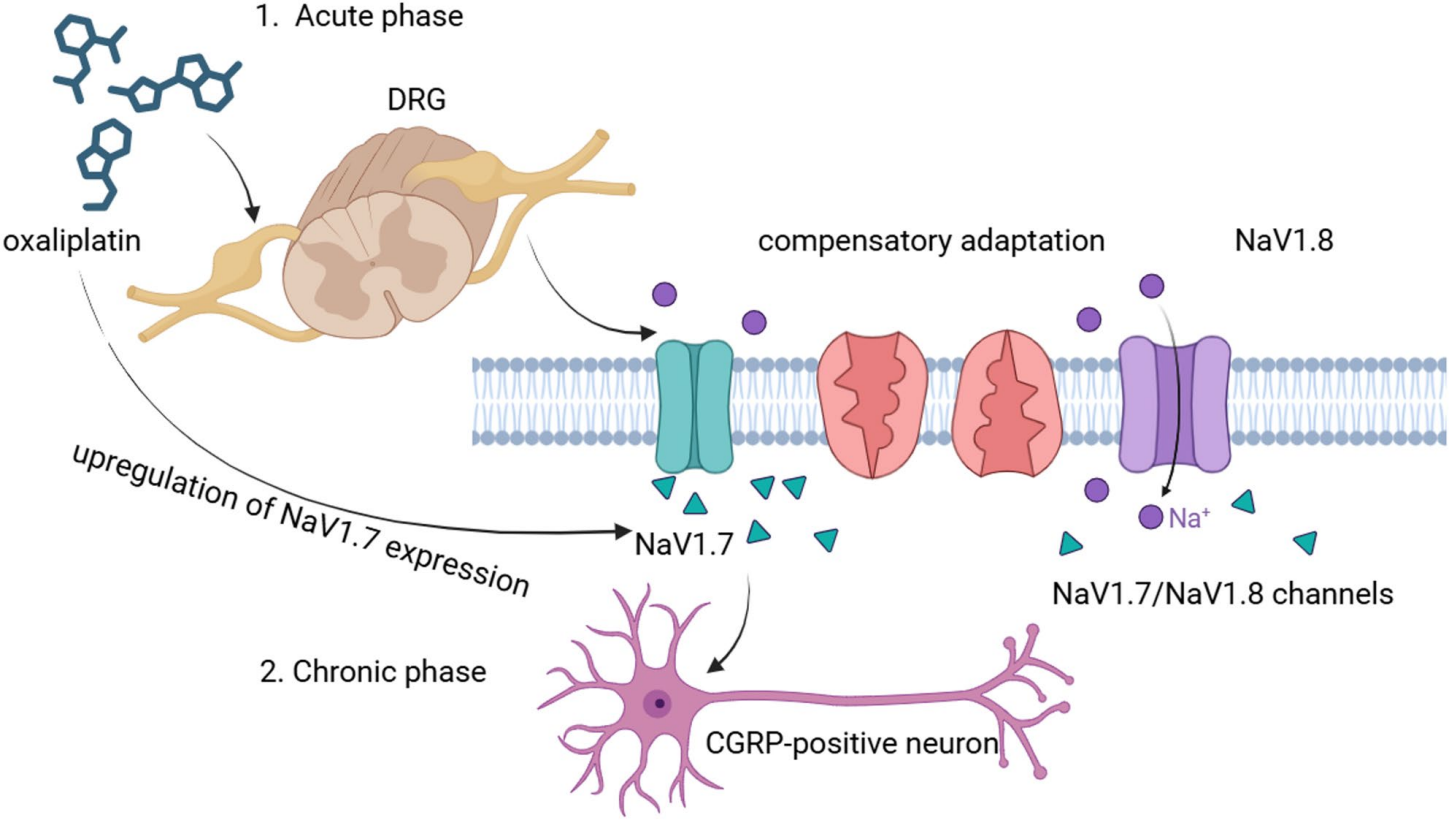
This schematic delineates the stage-specific regulation of voltage-gated sodium (NaV) channels in dorsal root ganglion (DRG) sensory neurons during oxaliplatin-induced peripheral neuropathy (OIPN). (1) Acute phase: Upon oxaliplatin exposure, compensatory adaptation of NaV1.7/NaV1.8 channels occurs in DRG sensory neurons, accompanied by slowed sensory conduction—consistent with the transient sensory abnormalities of acute OIPN. (2) Chronic phase: Prolonged oxaliplatin-mediated chemotoxicity drives upregulation of NaV1.7 expression, which is localized to projecting calcitonin gene-related peptide (CGRP)-positive neurons; this subtype-specific NaV1.7 upregulation contributes to the persistence of chronic neuropathic sensory phenotypes in OIPN

Early studies demonstrated that oxaliplatin rapidly enhances tetrodotoxin-resistant (TTX-R) sodium currents—predominantly mediated by NaV1.8—within minutes and alters their activation/inactivation kinetics, leading to transiently increased excitability of DRG neurons. This is considered the ionic basis for acute paresthesia [27, 28]. Recent studies have uncovered a more critical role of NaV1.7 in chronic OIPN. Animal experiments have shown that prolonged oxaliplatin exposure results in a significant upregulation of NaV1.7 (but not NaV1.8) mRNA and protein expression levels in the DRG [29]. Notably, this upregulation is particularly prominent in calcitonin gene-related peptide (CGRP)-positive neurons [30] -an important subset of peptidergic nociceptors that extensively project to the superficial laminae of the spinal dorsal horn. Overexpression and enhanced function of NaV1.7 in these neurons substantially lower the action potential threshold and increase firing frequency, thereby amplifying the transmission of pain signals to the central nervous system. Specifically blocking NaV1.7 channels can effectively reverse mechanical and cold hyperalgesia in OIPN model animals [31] (Fig. 2). These findings suggest that the selective upregulation of NaV1.7 by oxaliplatin is a key mechanism driving the maintenance of chronic neuropathic pain.

The dysfunction of ion channels is not an isolated event. Oxaliplatin may induce intracellular oxidative stress, alter membrane potential, or activate upstream signaling pathways (e.g., p38-MAPK as described below), which collectively contribute to the synergistic dysregulation of TRP and NaV channels, ultimately shaping the complex sensory symptom profile of OIPN.

### Sustained activation of the p38 mitogen-activated protein kinase (p38-MAPK) pathway: a signaling hub linking injury and sensitization.

p38-MAPK is a key signaling molecule in cellular stress responses and plays a central role in neuroinflammation and pain sensitization [32]. In the nervous system, p38 mediates neuropathic pain primarily in microglia and spinal astrocytes. However, in OIPN, the activation of p38 within primary sensory neurons (DRG) has attracted particular attention.

**Fig. 3:**
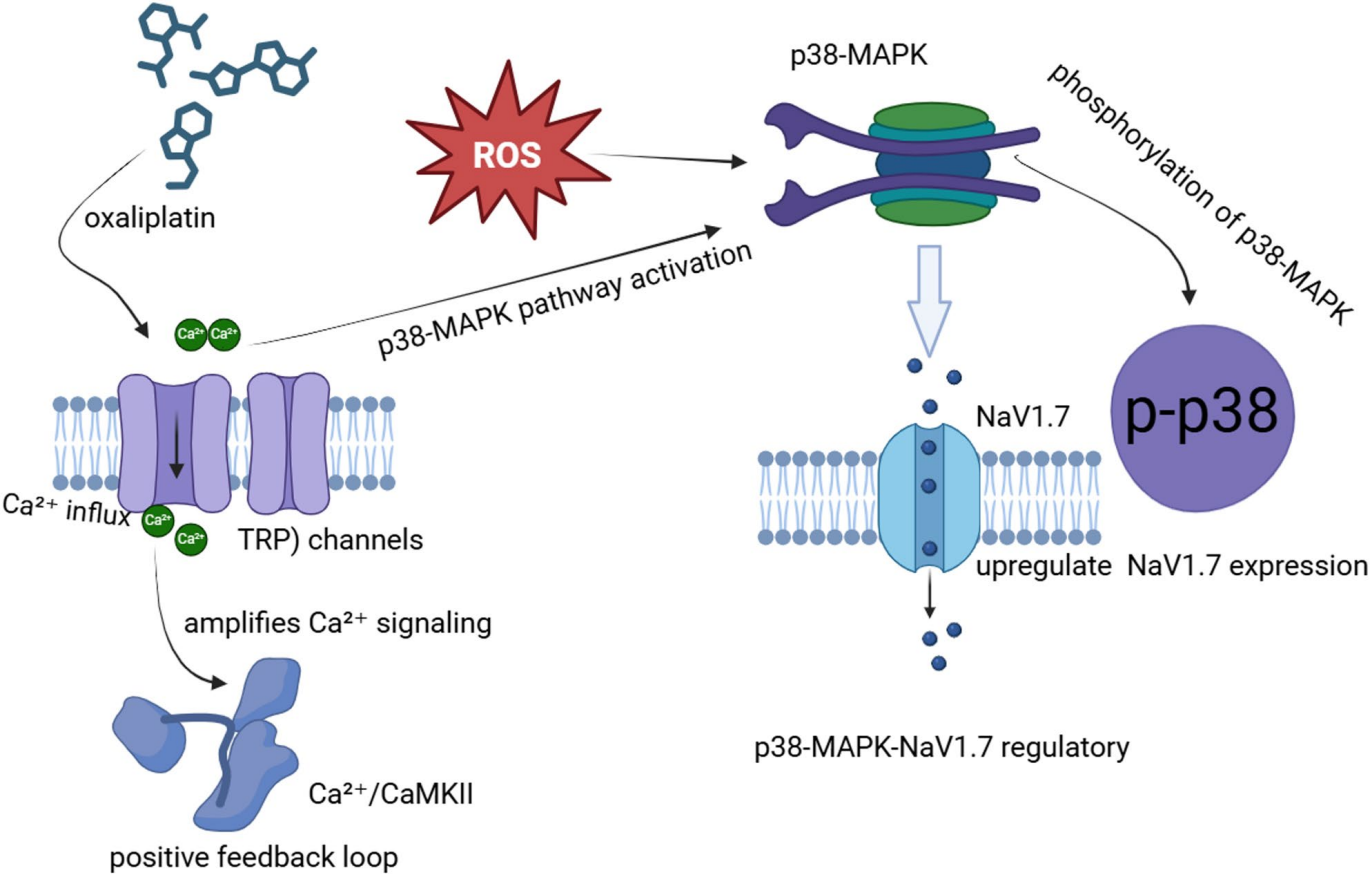
This schematic illustrates the multi-component regulatory network underlying neuronal sensitization in oxaliplatin-induced peripheral neuropathy (OIPN). (1) Initial Ca²⁺ signaling activation: Oxaliplatin acts on transient receptor potential (TRP) channels, triggering Ca²⁺ influx (Ca²⁺ flux). This Ca²⁺ signal activates Ca²⁺/calmodulin-dependent protein kinase II (CaMKII), forming a positive feedback loop that sustains and amplifies Ca²⁺ signaling. (2) p38-MAPK pathway activation: Ca²⁺ signals (or oxaliplatin directly) and reactive oxygen species (ROS) promote the phosphorylation of p38-MAPK (generating active p-p38), activating this stress-responsive signaling pathway. (3) p38-MAPK-mediated NaV1.7 regulation: Activated p38-MAPK functions as a hub: (i) it drives the transcription of NaV1.7 to upregulate its expression; (ii) it forms a p38-MAPK-NaV1.7 regulatory axis to further amplify pro-nociceptive signals

Both animal and cellular models have confirmed that oxaliplatin treatment significantly increases the level of phosphorylated p38 (p-p38, the active form) in DRG neurons [33]. This activation is also prominent in CGRP-positive neurons. Activated p38-MAPK regulates the expression of a series of pain-related genes by phosphorylating downstream transcription factors (e.g., ATF2, CREB) and modulating mRNA stability. Studies have shown that activation of the p38 pathway may be a critical intermediate link in oxaliplatin-induced upregulation of NaV1.7 expression in the DRG [34]. Inhibition of p38 activity not only reduces NaV1.7 expression but also attenuates oxaliplatin-induced increased neuronal excitability and behavioral hyperalgesia [35]. Additionally, p38 may directly modulate the function of channel proteins such as TRPV1 through phosphorylation [36]. Thus, the p38-MAPK pathway acts as a central processor, converting initial cellular damage signals induced by oxaliplatin (e.g., oxidative stress, DNA damage response) into persistent neuronal transcriptional reprogramming and electrophysiological remodeling, and serves as a key hub linking upstream injury to downstream ion channel abnormalities (Fig. 3).

### Dynamic remodeling of the dorsal root ganglion (DRG) immune microenvironment: from bystander to active participant

The traditional neurotoxicity paradigm views OIPN as direct neuronal damage induced by drugs. However, a growing body of evidence indicates that the dorsal root ganglion (DRG), a key structural component of the peripheral nervous system with relatively weak ‘immune privilege, harbors resident and infiltrating immune cells that exert active and complex roles in the initiation and progression of OIPN [37].

**Fig. 4:**
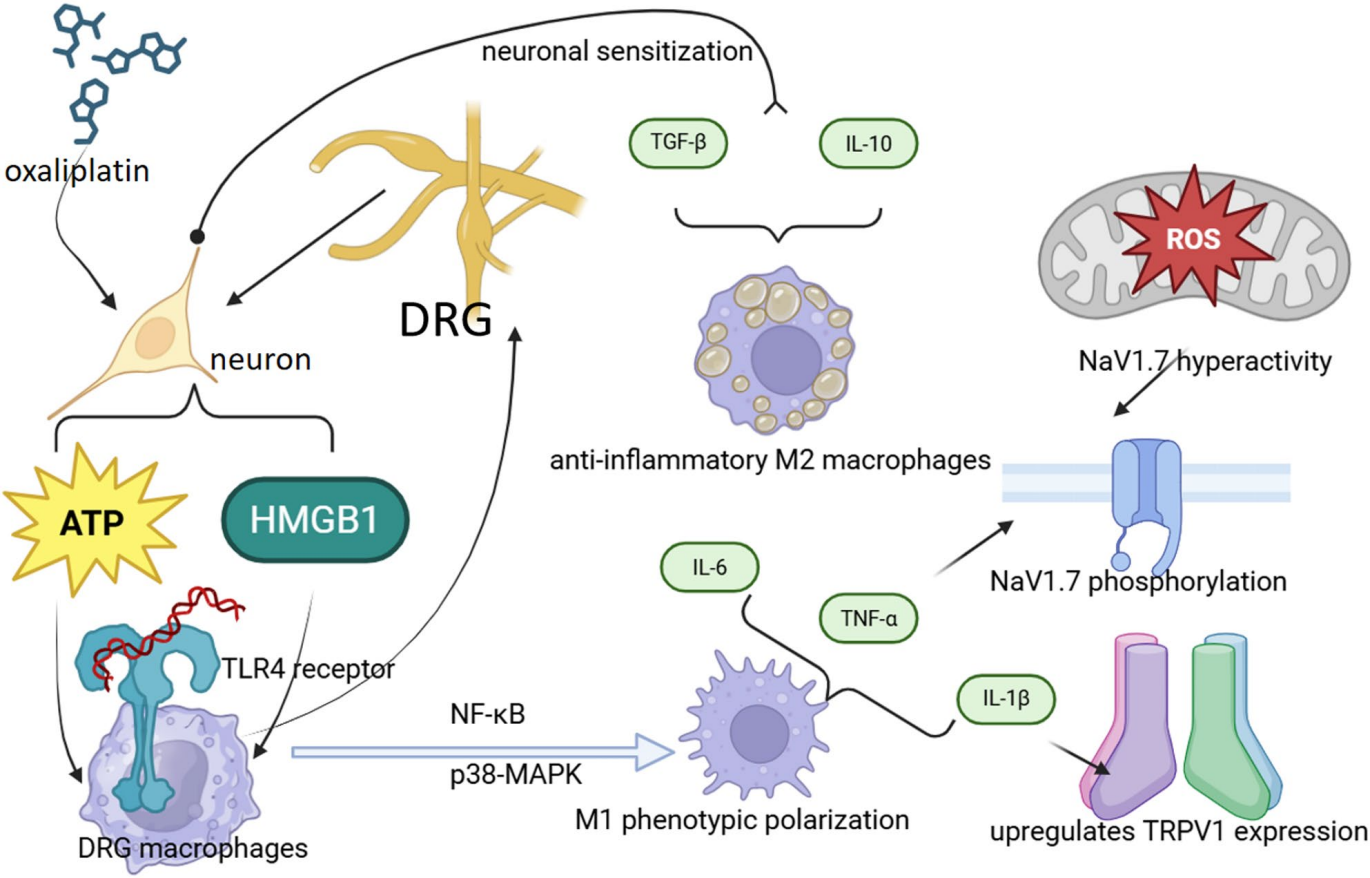
This schematic outlines the neuro-immune crosstalk network in dorsal root ganglion (DRG) mediating oxaliplatin-induced peripheral neuropathy (OIPN). (1) Initiation of neuro-immune signaling: Oxaliplatin-induced DRG neuronal injury triggers the release of damage-associated molecular patterns (DAMPs, e.g., ATP, HMGB1). These DAMPs activate resident DRG macrophages (via TLR4 receptor binding) and recruit peripheral macrophages to the DRG microenvironment. (2) Pro-inflammatory macrophage polarization: Activated macrophages undergo M1 phenotypic polarization via the NF-κB and p38-MAPK pathways, secreting pro-inflammatory cytokines (TNF-α, IL-1β, IL-6). (3) Cytokine-mediated neuronal sensitization: These pro-inflammatory mediators directly modulate sensory neurons: TNF-α promotes NaV1.7 phosphorylation to enhance its function; IL-1β upregulates TRPV1 expression; reactive oxygen species (ROS) also contributes to NaV1.7 hyperactivity. Collectively, these effects amplify pain signal transmission. (4) Dysregulation of pro-/anti-inflammatory balance: In OIPN, the proportion of anti-inflammatory M2 macrophages (which secrete IL-10, TGF-β) is reduced, disrupting the pro-/anti-inflammatory balance. The attenuation of M2-derived anti-inflammatory signals further exacerbates local neuroinflammation and neuronal sensitization

Resident macrophages exist in the DRG and can recruit circulating monocytes following injury. In OIPN models, the number of macrophages in the DRG is significantly increased and these cells become activated [38]. Macrophages exhibit high plasticity and are classified into pro-inflammatory M1 phenotype (secreting IL-1β, TNF-α, IL-6, etc.) and anti-inflammatory/repair M2 phenotype (secreting IL-10, TGF-β, etc.). Studies have demonstrated that oxaliplatin induces M1 polarization of DRG macrophages, leading to the release of large amounts of pro-inflammatory cytokines and chemokines [39]. These inflammatory mediators can directly activate or sensitize adjacent sensory neurons: For example, TNF-α rapidly enhances NaV1.7 and NaV1.8 currents, while IL-1β sensitizes TRPV1 channels [40, 41]. Meanwhile, signaling molecules such as CGRP and ATP released by neurons can reciprocally regulate macrophage function, forming a positive feedback loop of “neuro-immune crosstalk” that exacerbates local inflammation and neural sensitization [42] (Fig. 4). Depletion of macrophages or inhibition of their M1 polarization can alleviate OIPN symptoms to varying degrees [43].

**Fig. 5:**
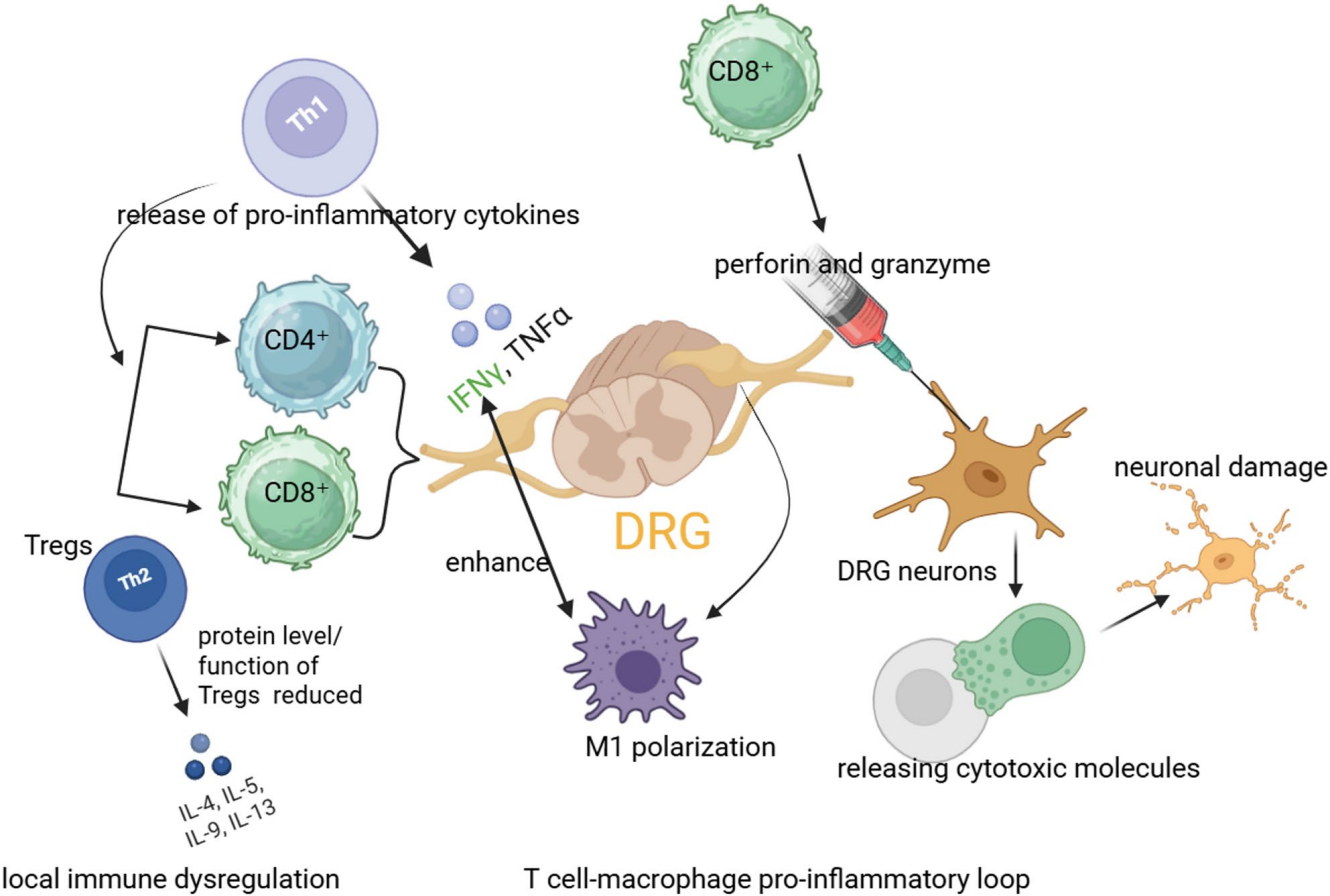
This schematic delineates the T cell-centered immunoregulatory network in the dorsal root ganglion (DRG) that mediates chronic oxaliplatin-induced peripheral neuropathy (OIPN). 1. T cell infiltration and subset imbalance: Infiltration of CD4⁺/CD8⁺ T cells (linked to Th1 cells) is elevated in the DRG, with concurrent release of pro-inflammatory cytokines (IFNγ, TNFα). While the proportion of Th2 cells increases, the protein level/function of regulatory T cells (Tregs) is reduced—this impairs immunosuppressive capacity and drives local immune dysregulation. 2. Direct neuronal modulation by CD8⁺ T cells: Infiltrated CD8⁺ T cells target DRG neurons via perforin and granzyme (e.g., releasing cytotoxic molecules), which may directly induce neuronal damage or sensitization. 3. T cell-macrophage pro-inflammatory feedback loop: IFNγ secreted by T cells enhances M1 polarization of DRG macrophages. This reciprocal interaction forms a T cell-macrophage pro-inflammatory loop, amplifying the local pro-inflammatory microenvironment

Recent studies have begun to focus on the role of T cells in OIPN (Fig. 5). Reports have shown that the numbers of CD4⁺ and CD8⁺ T cells are increased in the DRG and sciatic nerves of mice with chronic OIPN [44]. T cells may participate in the maintenance of neuroinflammation by releasing cytokines (e.g., IFN-γ, IL-17) or directly interacting with neurons/glial cells. Dysfunction of regulatory T cells (Tregs) may impair the inhibition of inflammation. However, the specific subsets, activation mechanisms, and functions of T cells in OIPN remain to be further explored. Mast cells, neutrophils, and other immune cells may also participate in the response during the early stages of OIPN. Additionally, satellite glial cells (neuronal support cells) within the DRG are also activated in OIPN, secreting inflammatory factors and contributing to neuronal sensitization [45].

Taken together, the pathogenesis of OIPN is a multidimensional, networked pathological process: Oxaliplatin first induces initial stress in DRG neurons, activating intracellular signaling pathways such as p38-MAPK. These pathways then regulate the expression and function of ion channels (e.g., upregulation of NaV1.7, sensitization of TRPA1), leading to neuronal electrophysiological dysfunction. Meanwhile, neuronal activation and release of damage signals (e.g., ATP, CGRP), in concert with the direct effects of oxaliplatin, remodel the DRG immune microenvironment and drive the shift of macrophages and other cells toward the pro-inflammatory phenotype. In turn, activated immune cells further sensitize neurons by releasing inflammatory mediators, forming a self-sustaining vicious cycle. This complex “neuron-immune crosstalk network” collectively contributes to the acute symptoms and chronic persistence of OIPN.

## Current preventive and therapeutic strategies for OIPN and their limitations

Due to the incomplete understanding of its pathogenesis, the prevention and treatment of oxaliplatin-induced peripheral neuropathy (OIPN) remain a major clinical challenge. Current strategies are primarily categorized into three types—prophylactic administration, symptomatic management, and neural repair—all of which have notable limitations.

### Mechanism-based preventive strategies for OIPN

Calcium-magnesium infusion was once hypothesized to reduce neurotoxicity by chelating oxaliplatin; however, large-scale randomized controlled trials (e.g., the CONcePT trial) have failed to demonstrate a clear long-term protective effect. Furthermore, it may compromise anti-tumor efficacy, and thus is not recommended for routine use in current clinical guidelines [46]. Antioxidants such as glutathione and alpha-lipoic acid are designed to counteract oxaliplatin-induced oxidative stress by directly scavenging reactive oxygen species (ROS) in peripheral tissues. While some small-scale studies have shown modest short-term protective effects against mild OIPN symptoms, their efficacy remains unconfirmed by large-scale Phase III randomized controlled trials, with inconsistent results across different cohorts [47].

### Symptomatic management agents

Duloxetine is the only agent recommended by the American Society of Clinical Oncology (ASCO) guidelines for the treatment of chemotherapy-induced peripheral neuropathic pain, based on a positive Phase III trial [48]. However, it primarily alleviates pain with limited efficacy on symptoms such as numbness, and is associated with adverse effects. Gabapentin and pregabalin are widely used, but have a relatively lower level of evidence supporting their efficacy [49]. Based on mechanistic research, inhibitors targeting NaV1.7 or TRPA1 are under development; however, they are still in preclinical or early clinical stages, with selectivity and systemic side effects remaining key challenges [50].

#### Ion channel modulators: suzetrigine for acute OIPN intervention

Suzetrigine, a recently FDA-approved selective antagonist of tetrodotoxin-resistant (TTX-R) NaV1.8 channels [51, 52], exhibits a well-characterized target profile on dorsal root ganglion (DRG) sensory neurons and has validated clinical efficacy and a favorable safety profile for the management of peripheral neuropathic pain. Its selective targeting of NaV1.8 aligns precisely with the core ionic mechanism of acute OIPN, as oxaliplatin acutely enhances NaV1.8-mediated TTX-R sodium currents in DRG neurons—the key ionic basis for acute paresthesia, cold hyperalgesia and abnormal neuronal excitability [53, 54]. Inhibition of NaV1.8 channels by suzetrigine could directly reverse this oxaliplatin-induced electrophysiological dysfunction, thereby exerting a targeted mitigating effect on the core acute neuropathic symptoms of OIPN.

Preemptive administration of suzetrigine prior to oxaliplatin infusion also holds theoretical potential for modulating the progression to chronic OIPN. Blocking acute NaV1.8-mediated neuronal hyperexcitability at the initiation of chemotherapy may abrogate the downstream pathological cascade triggered by early neuronal dysfunction, including oxidative stress overactivation, initial p38-MAPK pathway induction, and early perturbation of the DRG immune microenvironment. This intervention could potentially attenuate the persistent electrophysiological remodeling and molecular reprogramming of DRG neurons, thus reducing the incidence or severity of chronic cumulative OIPN.

Notably, direct preclinical in vivo evidence and clinical trial data validating suzetrigine’s efficacy, optimal dosing regimen, and drug-drug interaction profile for OIPN are currently lacking. Subsequent preclinical validation in OIPN animal models and early-phase clinical pilot trials are urgently needed to confirm its therapeutic potential when combined with oxaliplatin chemotherapy, and to clarify its clinical application value in OIPN management.

### Neural repair and regenerative therapy

Neurotrophic factors such as nerve growth factor (NGF) can promote neural repair and regeneration; however, NGF itself exhibits pronociceptive effects, limiting its clinical application [55]. Physical therapies, including exercise and cryotherapy, may exert modest adjuvant benefits but lack high-quality evidence to support their efficacy [56].

Overall, current therapeutic approaches are primarily symptomatic, failing to reverse or effectively prevent neural damage. The development of novel agents that can penetrate the blood-nerve barrier (BNB), target key pathological nodes in the DRG (e.g., ion channel dysfunction, p38-MAPK activation, immune dysregulation), and avoid compromising chemotherapy efficacy represents a key direction for future research. 

## Nanoparticle drug delivery systems: a promising yet challenging platform for overcoming key bottlenecks in OIPN therapy

Nanoparticle delivery systems (e.g., PLGA nanoparticles, liposomes, lipid nanoparticles) offer preclinical advantages for OIPN therapy: improving neuroprotective agent bioavailability, enhancing DRG penetration via the EPR effect, enabling stimulus-responsive release, and co-loading multi-mechanism drugs [57]. In OIPN therapy, the advantages of nanotechnology are mainly reflected in four aspects: (1) Improved bioavailability and stability: Encapsulation protects drugs (e.g., peptides, natural products) from degradation and prolongs their circulation time in the bloodstream. (2) Enhanced blood-nerve barrier (BNB) penetration: Nanoparticles can increase drug accumulation in peripheral neural tissues such as the DRG through passive targeting (enhanced permeability and retention [EPR] effect) or active targeting strategies (surface modification with ligands like transferrin receptor or choline) [58]. (3) Targeted delivery and controlled release: Drugs can be precisely delivered to specific cells (e.g., DRG neurons, M1 macrophages), and stimulus-responsive release at pathological sites can be achieved via material design, thereby improving therapeutic efficacy and reducing systemic toxicity [59]. (4) Co-drug loading: Nanocarriers can simultaneously load drugs with different mechanisms (e.g., anti-inflammatory agents + ion channel modulators) to achieve multi-targeted synergistic therapy.

Several studies have explored the protective effects of nanoparticles loaded with natural products (e.g., curcumin, resveratrol) in OIPN models, demonstrating superior efficacy compared to free drugs [60, 61]. However, this preclinical proof of concept does not constitute a clinical solution, as key translation challenges remain unaddressed previously. Critical practical challenges include: (1) Long-term carrier neurotoxicity: Prioritize biodegradable materials (e.g., PLGA-PEG copolymers) to avoid DRG accumulation-induced oxidative stress [62]; (2) In vivo stability: Mitigate MPS clearance via PEGylation or biomimetic coating (exosome/red blood cell membrane) [63, 64]; (3) Manufacturing scalability: Adopt GMP-compliant microfluidic fabrication for uniform batch production [65]. For selective targeting: CGRP-positive neurons are targeted via choline (CHT1 ligand) to mediate receptor-dependent endocytosis [66]; M1 macrophages are targeted via CSL362 (CD11b ligand) to enable selective binding and phagocytosis [67]. These validated ligands resolve the theoretical feasibility gap, ensuring specific delivery to pathological DRG cells. Dual-targeted nanocarriers (co-modified with choline and CSL362) synergistically intervene in ion channel dysfunction and neuroinflammation, aligning with OIPN’s multi-dimensional pathogenesis.

Notably, the advantages of nanoparticle systems—including DRG-targeted delivery, improved bioavailability, and multi-mechanism co-loading—directly address the unmet needs of OIPN therapy: overcoming biological barriers, targeting pathological cell subsets (CGRP+ neurons, M1 macrophages), and intervening in OIPN’s multi-dimensional pathogenesis. For natural neuroprotective agents with well-documented anti-inflammatory, antioxidant, and ion channel-modulating properties (e.g., salidroside), nanonization emerges as a pivotal strategy to unlock their therapeutic potential—resolving inherent limitations such as poor lipid solubility and inadequate BNB penetration that have hindered clinical application. The following section focuses on salidroside, whose multi-target pharmacological profile aligns with OIPN’s core mechanisms, and discusses how nanoparticle-mediated delivery can maximize its efficacy in OIPN.

## Salidroside: a promising natural neuroprotective agent and its nanonization prospects

### Pharmacological effects of salidroside

Salidroside (SAL), a major active component of Rhodiola rosea, exhibits a multi-target pharmacological profile that directly corresponds to the core pathophysiological mechanisms of OIPN—including oxidative stress, neuroinflammation, ion channel dysfunction (TRPV1/TRPA1/NaV1.7), mitochondrial injury, and immune microenvironment dysregulation (Sect.  2) [68]. This makes it a promising candidate for OIPN intervention, provided its delivery limitations (low oral bioavailability, poor BNB penetration) are resolved via the nanoparticle strategies outlined in Sect.  4. In models of neurological diseases, salidroside has demonstrated distinct neuroprotective effects: alleviating oxidative damage, inhibiting the excessive activation of microglia/astrocytes, reducing the release of pro-inflammatory cytokines, and promoting the expression of neurotrophic factors [69, 70]. In a peripheral neuroinflammation model, salidroside dually regulates neuroinflammation and immune homeostasis via the PI3K-AKT signaling pathway, ameliorating experimental autoimmune neuritis (EAN) [71]. SAL enhances Parkin-mediated mitophagy in the dorsal root ganglia (DRG) and alleviates peripheral nerve injury and pain. These findings indicate that Parkin-mediated mitophagy is involved in the pathogenesis of oxaliplatin-induced peripheral neuropathy (OIPN) [72]. In addition, SAL markedly promotes the proliferation and functional activity of Schwann cells (SCs), and its underlying mechanism may be attributed to the regulation of neurotrophic factors (BDNF, GDNF, and CNTF) by SAL to modulate SC growth [73, 74]. Furthermore, SAL can inhibit the activation of the TXNIP/NLRP3 inflammasome axis and alleviate nerve injury-induced neuronal loss in the dorsal horn of the spinal cord, thereby exerting significant analgesic and neuroprotective effects [75].

A critical yet under-elaborated mechanism of salidroside’s neuroprotective and analgesic action lies in its direct and indirect regulation of key pro-nociceptive ion channels in DRG sensory neurons—including voltage-gated sodium channels (NaV1.7, NaV1.8) and transient receptor potential (TRP) channels (TRPM8, TRPV1, TRPA1)—the core mediators of OIPN-associated neuronal hyperexcitability and pain signal transduction (Sect.  2.1). For voltage-gated sodium channels, salidroside may downregulates the mRNA and protein expression of NaV1.7 in DRG neurons via inhibiting the p38-MAPK/ATF2 signaling pathway [76], and attenuates oxaliplatin-induced enhancement of NaV1.8-mediated tetrodotoxin-resistant (TTX-R) sodium currents by reducing oxidative stress-mediated post-translational modification of channel proteins. This dual regulation directly elevates the action potential threshold of DRG neurons and reduces abnormal firing frequency, thereby inhibiting the amplification and transmission of OIPN-induced pain signals. For TRP channels, salidroside may blunts oxaliplatin-induced sensitization of TRPA1 and TRPV1 in DRG neurons by scavenging reactive oxygen species (ROS) and inhibiting NF-κB-mediated transcriptional upregulation [77]; it also suppresses TRPM8 overactivation and phosphorylation via downregulating the p38-MAPK pathway, directly targeting the core ionic mechanism of OIPN-specific cold hypersensitivity in human sensory neurons [72].

Notably, a recent mechanistic study has identified that salidroside significantly upregulates the expression of TASK1 (KCNK3), a two-pore domain potassium channel, in a subset of human DRG sensory neurons [78]. TASK1 is a constitutively active potassium channel that maintains the resting membrane potential of sensory neurons and modulates neuronal excitability; its downregulation is closely associated with increased DRG neuronal hyperexcitability in peripheral neuropathic pain models, including OIPN. Salidroside upregulates TASK1 expression via activating the PI3K-AKT/FOXO1 signaling pathway, which enhances potassium efflux from DRG neurons, hyperpolarizes the resting membrane potential, and reduces neuronal sensitivity to cold, mechanical, and chemical nociceptive stimuli [78]. This TASK1-mediated mechanism provides a novel and important molecular basis for salidroside’s analgesic effects: by upregulating this anti-nociceptive potassium channel, salidroside counteracts the pro-nociceptive effects of NaV and TRP channel dysfunction in OIPN, forming a “bidirectional regulation” pattern of inhibiting pro-nociceptive ion channels and activating anti-nociceptive ion channels in DRG neurons.

The ion channel-modulating effects of salidroside are highly synergistic with its other pharmacological properties in alleviating OIPN. Its antioxidant and anti-inflammatory effects reduce ROS-mediated ion channel modification and pro-inflammatory cytokine-induced channel sensitization, respectively, creating a favorable microenvironment for restoring normal ion channel function in the DRG; while its upregulation of TASK1 and downregulation of NaV1.7/NaV1.8 directly reverse DRG neuronal hyperexcitability—the core electrophysiological abnormality of OIPN. This multi-level, synergistic modulation of ion channels and the DRG microenvironment further solidifies salidroside’s mechanistic rationality as a candidate for OIPN therapy.

These properties are highly consistent with the core pathological mechanisms of OIPN (oxidative stress, neuroinflammation, increased neuronal excitability, mitochondrial injury, regulation of neurotrophic factors, and neuroregeneration), leading to the hypothesis that salidroside could represent a potential candidate for OIPN prevention and treatment—a premise that requires extensive preclinical validation in OIPN-specific models before any assessment of its clinical potential.

### The necessity and strategies for salidroside nanonization

However, salidroside exhibits high water solubility and poor lipid solubility, resulting in low oral bioavailability and difficulty in effectively penetrating biological barriers (including the blood-nerve barrier [BNB]) to reach its target site, the DRG [79]. Direct administration may fail to achieve effective therapeutic concentrations. Formulating it into nanoparticles (e.g., PLGA nanoparticles, lipid nanoparticles, polymer micelles) is an ideal strategy to address these issues. Nanonization can enhance its stability and biomembrane penetration capacity [80], promote DRG accumulation via passive or active targeting, and enable sustained release to prolong the duration of action.

To date, there are no published preclinical or clinical reports investigating the efficacy, safety, or mechanistic action of salidroside—either as a free compound or in nanoparticle formulations—for the prevention or treatment of OIPN. Existing research on salidroside-loaded nanoparticles is exclusively limited to brain disease models (e.g., ischemia-reperfusion injury) and tumor models [81, 82], with no translational data available for peripheral neuropathy of any etiology, let al.one OIPN.

In summary, salidroside’s multi-target properties directly address OIPN’s core pathological mechanisms (Sect.  2), while nanoparticle-mediated delivery (Sect.  4) resolves its inherent pharmacokinetic limitations. This ‘mechanistically aligned candidate + advanced delivery platform’ paradigm represents a rational, hypothesis-driven strategy for OIPN therapy—one that builds on the review’s foundational analysis of OIPN pathogenesis and delivery solutions.

## Integrating cutting-edge technologies: the application of spatial transcriptomics and single-cell sequencing in mechanistic research

To thoroughly elucidate the complex mechanisms of oxaliplatin-induced peripheral neuropathy (OIPN) and evaluate the efficacy of novel therapies, technologies capable of resolving molecular changes at both the tissue spatial architecture and single-cell resolution are required. The emergence of spatial transcriptomics (ST) and single-cell RNA sequencing (scRNA-seq) has made this feasible [83, 84]. Importantly, these technologies are not limited to mechanistic atlas mapping—they serve as a bridge to clinical solutions by identifying non-invasive surrogates for DRG pathology, avoiding the need for routine patient DRG biopsies.

Spatial transcriptomics can preserve in situ spatial location information within tissues while capturing whole-transcriptome data. When applied to the dorsal root ganglion (DRG), it enables precise analysis of gene expression differences across distinct functional regions, such as calcitonin gene-related peptide (CGRP)-high expression areas, immune cell infiltration zones, and vascular regions. This intuitively reveals the impact of oxaliplatin or drug intervention on the spatial molecular architecture of the DRG, as well as the positional information of cell-cell interactions [85].

Single-cell RNA sequencing (scRNA-seq) can unbiasedly identify all cell types in the DRG (e.g., neuronal subtypes, satellite glial cells, macrophages, T cell subsets, and endothelial cells) and uncover dynamic transcriptomic changes, novel biomarkers, and signaling pathways of each cell type during OIPN progression [86]. In particular, it can finely resolve the heterogeneity of the immune microenvironment—such as distinguishing M1/M2 macrophages and T cell subsets with distinct functions—and infer their intercellular communication networks.

Combined application of these two technologies to DRG samples from OIPN models and those treated with salidroside-loaded nanoparticles is expected to achieve the following breakthroughs: (1) Mapping high-resolution spatiotemporal molecular atlases of OIPN development; (2) Precisely localizing the primary target cell types and spatial regions of salidroside-loaded nanoparticles; (3) Systematically elucidating the molecular networks through which salidroside regulates ion channels, the p38-MAPK pathway, and immune cell phenotypic switching at the single-cell level; (4) Identifying DRG-specific pathological gene signatures that are mirrored in accessible tissues (peripheral blood mononuclear cells [PBMCs], skin biopsies)—a critical step for clinical translation.

A key clinical application enabled by these atlases is the development of non-invasive biomarkers for OIPN management. For example, scRNA-seq can identify a unique gene expression signature of CGRP+ neurons with NaV1.7 upregulation (a core driver of chronic OIPN) or M1 macrophages secreting IL-1β/TNF-α. ST further validates the spatial co-localization of these cells with pathological events. These signatures can then be validated in PBMCs or skin biopsies from OIPN patients [87, 88], eliminating the need for DRG biopsies.

In clinical practice, this allows: (1) Pre-treatment prognosis: Patients with high levels of the M1 macrophage signature in PBMCs can be identified as high-risk for chronic OIPN and prioritized for preventive intervention with salidroside nanomedicines; (2) Therapy selection: Patients with the NaV1.7/CGRP neuronal signature are more likely to respond to salidroside-based therapies targeting ion channels; (3) Response monitoring: Serial PBMC testing of these signatures can non-invasively assess whether salidroside nanomedicines effectively reverse DRG pathology during treatment.

For the hypothetical salidroside-loaded nanomedicines, these technologies provide critical clinical translation data: validating target engagement in preclinical models, identifying patient subgroups most likely to benefit, and developing non-invasive biomarkers to guide clinical trial design and routine patient care. This directly addresses the clinical dilemma of limited effective OIPN therapies by translating mechanistic insights into actionable precision medicine strategies. This will attain a depth and breadth that is unattainable with traditional molecular biology methods.

## Discussion

This section synthesizes the core concepts of oxaliplatin-induced peripheral neuropathy (OIPN) mechanisms, therapeutic strategies, and technological advancements, while highlighting current limitations, translational challenges, and future research directions to provide an integrated perspective.

### Current limitations in OIPN mechanism elucidation

Despite significant progress, the understanding of OIPN pathogenesis remains incomplete. First, the heterogeneous responses to oxaliplatin among patients suggest uncharacterized genetic or epigenetic modifiers that regulate ion channel dysfunction (e.g., NaV1.7/TRPA1) and immune microenvironment remodeling, which current animal models fail to fully recapitulate. Second, the spatial-temporal dynamics of “neuron-immune crosstalk” in the dorsal root ganglion (DRG) are not fully defined—how M1 macrophages and T cells precisely interact with CGRP-positive neurons to sustain neuroinflammation requires further investigation. Third, the interplay between mitochondrial injury, mitophagy, and p38-MAPK pathway activation remains unclear, limiting the identification of core regulatory nodes.

### Translational challenges in therapeutic development

The translation of preclinical findings to clinical practice faces multiple bottlenecks. For nanomedicine, long-term neurotoxicity of carrier materials, in vivo stability (e.g., PEG dilemma), and scalable manufacturing hinder clinical translation, despite promising preclinical results. Current therapies are predominantly symptomatic, and mechanism-based agents (e.g., NaV1.7 inhibitors) lack selectivity, leading to systemic side effects. Additionally, salidroside, while exhibiting multi-target neuroprotective properties, lacks OIPN-specific preclinical validation, and its nanonization requires optimization of DRG-targeting ligands and stimulus-responsive release systems to ensure efficacy and safety.

### Future research directions

To address these gaps, future studies should focus on three key areas: (1) Leveraging spatial transcriptomics and single-cell RNA sequencing to decode DRG molecular heterogeneity and identify conserved, non-invasive biomarkers (e.g., PBMC-derived signatures) for patient stratification. (2) Optimizing dual-targeted nanocarriers (co-modified with CHT1 and CD11b ligands) to deliver salidroside and synergistic agents (e.g., p38-MAPK inhibitors), addressing OIPN’s multi-dimensional pathogenesis. (3) Conducting systematic preclinical studies to validate salidroside’s efficacy in OIPN models, while evaluating its interaction with chemotherapeutic agents to avoid compromising anti-tumor efficacy. These efforts will advance precision prevention and treatment strategies for OIPN.

## Conclusions and future perspectives

Oxaliplatin-induced peripheral neuropathy (OIPN) is a complex pathological process co-driven by abnormal sensory neuron ion channel function, sustained activation of key signaling pathways (e.g., p38-MAPK), and dynamic remodeling of the dorsal root ganglion (DRG) immune microenvironment. Among these, TRPA1-mediated acute cold hyperalgesia, NaV1.7 upregulation (particularly in CGRP+ neurons)-driven chronic hyperexcitability, and immune cell (e.g., macrophage)-mediated neuroinflammation constitute the three core pillars of its pathogenesis. Despite advancing mechanistic insights, clinical translation remains lagging. Nanotechnology-based drug delivery systems offer promise for overcoming the delivery barriers of neuroprotective agents. Combining salidroside, a natural product with well-documented multi-target neuroprotective properties in other neurological disease models, with nanotechnology to develop salidroside-loaded nanoparticles constitutes a hypothetical research direction that merits systematic preclinical investigation, given the complete absence of empirical evidence for salidroside’s effects in OIPN models of any species. Furthermore, leveraging cutting-edge technologies such as spatial transcriptomics (ST) and single-cell RNA sequencing (scRNA-seq) not only enables the elucidation of OIPN pathogenesis with unprecedented precision but also provides a rigorous experimental framework to explore the potential efficacy and mode of action of salidroside-loaded nanomedicines—laying a theoretical foundation for the future exploration of precision prevention and treatment strategies for OIPN, should preclinical validation of this hypothetical approach be achieved.

Future research should focus on the following aspects: (1) Optimize the design of salidroside-loaded nanoparticles with DRG-targeting capability and intelligent drug release properties; (2) Systematically validate their efficacy and safety for OIPN prevention and treatment in preclinical models; (3) Comprehensively utilize multi-omics technologies to elucidate the integrated mechanisms by which salidroside exerts its effects—via regulating ion channels, inhibiting the p38-MAPK pathway, and remodeling the immune microenvironment (particularly macrophage/T cell balance)—at the spatial and single-cell levels; (4) Explore whether their combination with chemotherapeutic agents impacts anti-tumor efficacy. Through these targeted preclinical efforts to validate this hypothetical strategy, it may ultimately yield new preventive and therapeutic options for cancer patients suffering from OIPN—an outcome that would hold significant scientific significance and clinical value, if the proposed mechanistic hypotheses are experimentally confirmed.

## Data Availability

Not applicable.
